# The Use of Indocyanine Green (ICG) and Near-Infrared (NIR) Fluorescence-Guided Imaging in Gastric Cancer Surgery: A Narrative Review

**DOI:** 10.3389/fsurg.2022.880773

**Published:** 2022-06-28

**Authors:** Francesco Belia, Alberto Biondi, Annamaria Agnes, Pietro Santocchi, Antonio Laurino, Laura Lorenzon, Roberto Pezzuto, Flavio Tirelli, Lorenzo Ferri, Domenico D’Ugo, Roberto Persiani

**Affiliations:** ^1^ Università Cattolica del Sacro Cuore, Rome, Italy; ^2^ Dipartimento Di Scienze Mediche E Chirurgiche, Fondazione Policlinico Universitario Agostino Gemelli IRCCS, Rome, Italy

**Keywords:** indocyanine Green, ICG, near-infrared fluorescence imaging, near-infrared, nir, gastric cancer

## Abstract

Near-infrared fluorescence imaging with indocyanine green is an emerging technology gaining clinical relevance in the field of oncosurgery. In recent decades, it has also been applied in gastric cancer surgery, spreading among surgeons thanks to the diffusion of minimally invasive approaches and the related development of new optic tools. Its most relevant uses in gastric cancer surgery are sentinel node navigation surgery, lymph node mapping during lymphadenectomy, assessment of vascular anatomy, and assessment of anastomotic perfusion. There is still debate regarding the most effective application, but with relatively no collateral effects and without compromising the operative time, indocyanine green fluorescence imaging carved out a role for itself in gastric resections. This review aims to summarize the current indications and evidence for the use of this tool, including the relevant practical details such as dosages and times of administration.

## Introduction

Gastric cancer is the fourth malignancy worldwide and the fourth cause of cancer-related deaths, with an incidence of 5.6% among new cancer diagnoses (1).

Surgery remains the cornerstone of curative-aim treatment, and the implementation of surgical quality represents one of the main branches of research on this topic. During the last two decades, intraoperative navigation tools have been introduced in various aspects of oncosurgery to improve the quality of care. One of the emerging technologies with the widest diffusion is near-infrared (NIR) fluorescence imaging with indocyanine green (ICG). ICG was approved for the first time in clinical practice by the FDA more than 50 years ago; however, its use has significantly expanded with the development of minimally invasive surgery. In fact, thanks to specific visualization tools such as NIR cameras, new possibilities for augmented visualization have been opened, which allow for real-time, high-definition visualization by switching between different modalities to visualize the indocyanine green fluorescence with the same laparoscope. Recently, laparoscopic surgery has been validated as a safe option for the treatment of gastric cancer in both Eastern and Western countries (2–14). Results of trials that investigate the non-inferiority of this technique in treating advanced gastric cancer are ongoing, but it is foreseen that mini-invasive approaches to gastrectomy will be pursued. The aim of this study was to summarize the current indications and evidence on the use, dosage, and timing of ICG administration in gastric cancer surgery, summarize the current evidence on the topic to familiarize gastric surgeons with this technology, and identify the gaps in knowledge to guide future research.

## ICG - Pharmacokinetics and Pharmacodynamics

ICG is a water-soluble compound with a molecular weight of 774.96g/mol. It is a tracer that emits fluorescence when stimulated by near-infrared light or a laser beam, and it possesses a maximum absorption wavelength of 800–820nm in water. Once injected into the human body, it immediately binds to plasma proteins, is processed in the liver by hepatocytes, and is then excreted into the bile juice. Its half-life is less than 3min. Because of the high protein content in the lymph, the bound ICG accumulates in the lymphatic system and highlights node stations prior to being metabolized by the liver. ICG reaches its maximum fluorescence when it binds to plasma proteins at a wavelength of 840nm in the blood. It has been recently shown that ICG preferentially binds to alpha-1 lipoprotein more than albumin, as traditionally reported; additionally, high-density lipoproteins are the most involved in ICG fluorescence (15). The scope adopted to capture the fluorescence uses both infrared light at wavelengths greater than 800nm and light-emitting diode (LED) light at a wavelength of 760nm; a filter is then used to switch to fluorescence mode, excluding lights below 820nm, thus allowing the tracer in the tissue to be visible (16). Fluorescence intensity does not relate to ICG concentration in a linear fashion; instead, it increases in the low concentration range of ICG, peaks, and then decreases with higher concentrations. This phenomenon has been described as the “quenching effect” (15). With regard to the dosages of the injections, there were no fixed rules: ICG powder in vials is diluted in sterile water at different concentrations and then administered directly to the four cardinal points around a tumor or directly in the serosa. When used intravenously, the dose of an ICG powder solution diluted in sterile water is usually 0.2–0.5mg/kg. The dosage can also be affected by the timing and site of injection. There is no standard rule for the time of injection, which varies from the day before to 20min before dissection (Table 1). The safety dose has been established between 0.025 and 0.5mg/kg; adverse reactions, including nausea, fever, and shock have been reported with a dose over 0.5mg/kg (17). Intraoperative injection may be preferable in terms of visualization of the lymphatics and the related guide functions during laparoscopic procedures, without spillage. This could also be a safer solution in cases of anaphylaxis (18).

**Table 1:** Summary of included studies.

	Aim	Time of injection	Injection point	Dosage
Yano K. et al. (2012) (19)	Identify the sentinel lymph node	After the surgical incision	Tumor marking at 4 sites Endoscopical submucosal injection	0.5 mL of a 0.5 mg/mL solution
Ushimaru Y. et al. (2019) (21)	Determining the tumor location	1 day before	Tumor marking at 4 sites Endoscopical submucosal injection	1 mL of a 0.05 mg/mL solution
Romanzi A. et al. (2021) (22)	Visualization of draining nodes	18h before surgery	Tumor marking at 4 sites Endoscopical submucosal injection	0.6 mL of a 1.25 mg/mL solution
Chen Q. et al. (2020) (23)	Visualization of draining nodes	1 day before	Tumor marking at 4 sites Endoscopical submucosal injection	0.5 mL of a 1.25 mg/mL solution
Kwon IG. et al. (2019) (24)	Visualization of draining nodes	1 day before	Tumor marking at 4 sites Endoscopical submucosal injection	0.6 mL of a 1.25 mg/mL solution
Kim T. et al. (2018) (25)	Visualization of draining nodes	15min before dissection	Tumor marking at 4–5 sites Endoscopical submucosal injection	1 mL of a 0.05 mg/mL solution
Park J. et al. (2021) (26)	Visualization of draining nodes	After surgical incision	20–25 sites along the greater and lesser curvatures Intraoperative administration by the surgeon	0.5 mL of a 0.025 mg/mL solution
An JY. et al. (2020) (31)	Identify the sentinel lymph node	During surgery	Tumor marking at 4 sites Endoscopical submucosal injection	1 mL of a mixed solution (2 mL of 2.5 mg/mL ICG and 2 mL of 99mTc-radiolabelled human serum albumin)
Miyashiro I. et al. (2011) (33)	Identify the sentinel lymph node	During surgery	Tumor marking at 4–8 sites Endoscopical submucosal injection	2–4 mL of a 0.25–1.25 mg/0.5 mL solution
Miyashiro I. et al. (2014) (34)	Identify the sentinel lymph node	During surgery	Tumor marking at 4 sites Intraoperatively serosal injection	4–5 mL of a 5 mg/mL solution
Roh CK. et al. (2020) (36)	Assess the completeness of the lymphadenectomy	1 day before	Tumor marking at 4 sites Endoscopical submucosal injection	0.6mL of a 1.25 mg/mL solution (da Vinci® Si) 0.6mL of a 0.625 mg/mL solution (da Vinci® Xi)
Tajima Y. et al. (2010) (40)	Visualization of draining nodes	1 to 3 days before the operation or during surgery	Tumor marking at 4 sites Endoscopical submucosal injection and intraoperatively serosal injection	0.5 mL of a 0.5% solution
Lan Y. et al. (2017) (56)	Visualization of draining nodes	Intraoperative and then 1 day before surgery	Tumor marking at 4 sites Intraoperatively serosal injection then endoscopical submucosal injection	0.6 mL of a 2.5 mg/mL solution
Cianchi F. et al. (2020) (58)	Visualization of draining nodes	1 day before	Tumor marking at 4 sites Endoscopical submucosal injection	0.5 mL of a 1.25 mg/mL solution
Chen Q. et al (2021) (59)	Visualization of draining nodes	1 day before VS 20 min before dissection	Tumor marking at 4 sites with endoscopical submucosal injection vs. intraoperatively at six specific sites along the lesser and greater curvature of the stomach	0.5 mL of a 1.25 mg/mL solution vs. 1.5 mL of a 0.5 mg/mL solution
Hunag Z. et al (2021) (61)	Visualization of draining nodes	After preoperative exploration	Intraoperatively at six specific sites along the lesser and greater curvature of the stomach	1.5 mL of a 0.5 mg/mL solution
Lee S. et al (2021) (33)	Visualization of draining nodes	1 day before	Tumor marking at 4 sites Endoscopical submucosal injection	0.6 mL of a 1.25 mg/mL solution (da Vinci® Si) 0.6 mL of a 0.625 mg/mL solution (da Vinci® Xi)
Huh Y. et al (2019) (62)	Perfusion of anastomosis	Immediately after anastomosis	Intravenous administration	1–2 mL bolus of a 2.5 mg/mL solution
Mori M. et al (2020) (69)	Perfusion of anastomosis	Immediately after anastomosis	Intravenous administration	Bolus of a 0.5 mg/kg solution

## Search Strategy

The electronic databases PubMed, Scopus, Web of Science, Cochrane Library, and clinicaltrials.gov were searched from May 2021 to January 2022 for papers inherent to the topic of this review. The search terms were “gastric cancer,” “near-infrared,” “near-infrared imaging,” “near-infrared fluorescence imaging,” “indocyanine green,” and “ICG.” Abstracts were selected, and the full text was evaluated. Articles were considered according to their level of evidence, timeliness, and ability to influence the current treatment for gastric cancer.

## Summary of the Applications in Gastric Surgery

In gastric cancer surgery, NIR with ICG has been employed according to the surgical procedure and technique required and the clinical stage of the disease. Initially, it was used for the detection of sentinel lymph nodes in early gastric cancer (19, 20). Its use has then been extended to preoperative endoscopic marking to assess tumor location, with the aim of achieving negative resection margins (21) and node mapping during D2 gastrectomy (22–22). The technique has also proven to be relevant in detecting anatomical structures, considering the complex anatomy of the perigastric vessels; it is also used in esophagogastric junction (EGJ) and esophageal tumors for the assessment of good perfusion of the anastomosis. Although the use of NIR with ICG has been increasingly popularized for laparoscopic gastrectomy, it allows for the performance of multiple real-time intraoperative evaluations in open procedures (26).

## Lymph Nodes

### Sentinel Node Navigation

The sentinel lymph node (SLN) is the first draining node from a primary neoplasm, and if the first draining node is considered negative for metastasis, all others are assumed to be negative as well. Different types of tracers have been proposed over the last decade, including radioisotopes and dyes (99mTc, sulfan blue, and isosulfan blue), for the effective detection of sentinel nodes in gastric cancer. In a 2013 prospective multicenter trial, a group from the Japan Society of Sentinel Node Navigation Surgery (SNNS) suggested a dual tracer submucosal injection with a radioisotope (technetium 99 m–labeled tin colloid) and 1% isosulfan blue dye. The technique was shown to be safe and effective in detecting sentinel lymph nodes in cT1 and cT2 tumors smaller than 4cm. A total of 397 patients underwent sentinel node biopsy, and the method showed high accuracy in detecting sentinel nodes and metastatic sentinel nodes, with a false negative rate of 1% (27).

Recently, near-infrared (NIR) ICG fluorescence has been proposed as a solution to overcome the factors associated with radioactive tracers or dyes, including its cost, iatrogenic effects, and ease of use. Notably, the first use of ICG was for the detection of sentinel lymph nodes in early gastric cancer (28); since then, its use has expanded tremendously. Given its good tropism for lymphoadipose tissue and the low incidence of allergic reactions, it has been considered preferable to other traces such as sulfan blue. In a 2014 systematic review and meta-analysis, Xiong et al. described an improvement in the detection rate of sentinel nodes and in sensitivity with the use of ICG as an alternative to conventional tracers after 2012, despite the relative heterogeneity among the studies considered. This trend in ICG use was judged positively in terms of technological advancement, and the results of future studies are anticipated. In terms of ICG guided sentinel node biopsy results in the detection of gastric cancer, the detection rate was 100%, despite a sensitivity of 84% (29). The existing literature documents a positive trend in the data reported over the last decade, which may reflect improvements in the technique.

In 2020, a systematic review and meta-analysis by Huang et al. indicated that compared with blue dye or radiocolloid tracer, ICG exhibited increased identification rates over time, as well as increased sensitivity and negative predictive values (NPVs of 98%, 88%, 96% in 2001–2010 to 99%, 92%, 98% in 2011–2020). The review suggested some theoretical advantages related to the use of both NIR ICG fluorescence alone and in combination with a dual-tracer method (radiocolloid + ICG) that allows objective measures. The authors exposed concerns regarding the costs, risks, and logistics associated with the use of radioactive substances. Therefore, the ICG technique could be considered the preferred technique by experts (30).

In the SENORITA clinical trial from the Korean National Cancer Center, the SEntinel Node ORIented Tailored Approach study group randomized 580 patients with cT1 N0 tumors that were <3cm in size and compared the results of laparoscopic stomach-preserving surgery and sentinel lymph node basin dissection (LSNNS) versus laparoscopic standard gastrectomy (LSG) after the injection of 99mTc-radiolabelled human serum albumin and indocyanine green. The trial also performed an intraoperative pathological examination protocol, including immunohistochemistry and molecular biological techniques, which are crucial for the detection of metastatic lymph nodes. Results from the trial suggest that the dual-tracer method is superior to ICG alone for successful laparoscopic sentinel node navigation surgery. The 30-day complication rates were similar in the LSNNS and LSG groups. The authors recommend precise preoperative evaluation of both patient and tumor characteristics in order to improve the success rate of the procedure. Maximum attention must also be paid intraoperatively to guarantee adequate perfusion and innervation of the remnant stomach to ensure a safe organ-sparing procedure. In terms of 3-year disease-free survival, the primary endpoint of the trial, LSNNS did not show non-inferiority compared to LSG. However, the 3-year overall survival and 3-year disease-specific survival rates were comparable, leaving a place for sentinel node navigation surgery in clinical practice (31, 32). More studies are needed to demonstrate the efficacy and safety of this approach in terms of patient quality of life and the risk of recurrence. Currently, most authors tend to administer injections for sentinel lymph node navigation in locations that are proximal, distal, and lateral to the tumor. This type of cardinal pattern is sufficient for most neoplasms, particularly early gastric cancer; in addition, if needed, additional injections can be administered for larger tumors (33). Injections can be subserosal, performed by a surgeon, or submucosal, performed by an endoscopist. Once detected, the sentinel lymph node must be removed “en bloc” with the corresponding lymph node basin according to the Japanese classification of gastric node stations. This could be considered the standard sentinel node biopsy technique. In SNNS papers, the definition of a basin can vary: it can refer to stations along the five principal arteries supplying the stomach or to limited areas specifically described, typically comprising lymphatic routes flowing from the tumor.

To obtain high-quality data, the Gastric Cancer Surgical Study Group (GCSSG) published a multicentric prospective clinical trial (JCOG0302) that aimed to evaluate the feasibility and accuracy of sentinel node evaluation in T1 gastric tumors smaller than 4cm. The protocol called for the injection of indocyanine green into the subserosal layer in proximity to the neoplasm. The rate of green nodes detected was high (97.8%), but there was an unexpected incidence of false negative cases (46.4%) (i.e., negative for metastasis in the intraoperative frozen section but positive during the definitive pathological evaluation). Subsequently, the trial was terminated. One of the main concerns was attributed to the single plane intraoperative histological examination performed on the bioptic material, which determined to not be proficient. The study aim was to demonstrate the feasibility of the intraoperative examination, but a large number of green, negative nodes were intraoperatively found to be metastatic *via* finally paraffin sectioning, misleading the surgeon in whether to perform a gastrectomy or not. Another limitation of the study was related to the learning curve, which was too short and could not be evaluated due to the termination of the trial (34). Compared to the SNNS trial, the JCOG0302 trial included 30 hospitals that contributed 5 patients each, while the former included 12 hospitals with at least 30 cases of experience. According to the existing literature, the learning curve allowing a success rate of 95% for sentinel node dissection could be attested in 26 cases (35). ICG fluorescence lymphography has also been studied after endoscopic submucosal dissection (ESD) when the specimen revealed a neoplasm classified as Sm > 1 and was therefore treated with gastrectomy. ICG was proficient in identifying lymphatic drainage, despite the scars produced by the endoscopy, suggesting that SNNS is still possible after ESD. The study, while considering the concerns regarding SNNS after ESD, also evaluated the drainage patterns during gastrectomy: they noted 100% sensitivity and 100% NPV for the detection of lymph node (LN) metastasis in the fluorescent station. No metastatic nodes were outside the highlighted area, suggesting that more targeted lymphadenectomy should be considered in this particular setting (36).

### Drawbacks to SNNS

The main concerns after performing SNNS are false-negative results and skip metastasis. False negatives are usually due to larger and more invasive tumors that have higher rates of lymph metastases, with approximate rates of 5%, 20%, and 50% respectively for T1a, T1b and T2 tumors. These proportions increase for early gastric cancers greater than 4cm (37). In fact, ICG fluorescence could indicate drainage of the tissue surrounding the tumor but not metastatic nodes, as well as the chance of “false-negatives.” This phenomenon could be due to the obstruction of the lymphatics caused by cancer cells. Consequently, the tracer moves to the second-tier node. This is the reason why the sentinel lymph node technique is not considered by some authors to be appropriate for gastric tumors >T1. To date, false negative rates vary between 23.5% and 60% (38–42). Therefore, to reduce false negatives, some authors have suggested exploring stations 7, 8, and 9 if the sentinel node is not found in the perigastric stations (43). Attempts have been made to reduce false negative rates with the development of new technologies such as nucleic acid amplification, which directly amplifies the mRNA of the molecular marker used in the supernatant of homogenized lymph nodes, with the aim of improving the sensitivity offered by hematoxylin and eosin intraoperative analysis (44–46). Other attempts included reverse transcription polymerase chain reaction (RT-PCR) (47, 48) and immunohistochemistry (49). On the other hand, skip metastasis (i.e., the presence of second-level positive stations with negative perigastric stations) may be a major concern in sentinel node navigation for gastric cancer among both Eastern (when evaluating T1–2 cancers) and Western institutions (when evaluating more advanced tumor stages) (50–52). Skip metastases reflect the tumor's location. For gastric tumors located in the lesser curvature, particularly in the lower part or circumferentially, there is a higher risk of skip metastasis, with a reported incidence up to 11% (53). Given the relatively low incidence of lymph node metastasis from early gastric cancer, studies collecting thousands of cases may be required to obtain high-quality data.

### Lymph Node Mapping

ICG has also been tested as an effective guide for LN dissection during standard gastrectomy. Harvesting a sufficient number of lymph nodes is essential for proper staging. Most guidelines recommend retrieval of a minimum of 16 regional nodes for pathological examination, and it remains desirable to collect 30 or more nodes (54, 55). In this setting, ICG lymph node mapping is intended to recognize lymph nodes and lymphatics and to help perform complete lymphadenectomy for the selected procedure. Small-sample retrospective studies were conducted to evaluate the effectiveness of ICG-guided lymphadenectomy. These studies reported non-unique results and highlighted some limitations, including difficulties in resecting fluorescent stations that are usually not included in standard D2 lymphadenectomy (i.e., stations 13, 14v, or 16a). In contrast, some studies have shown a greater number of retrieved nodes in the critical stations of laparoscopic procedures that use NIR ICG (22, 25, 39, 56).

A recent prospective study by Kwon et al. compared the results from performing robotic gastrectomy in stage I gastric cancer patients that underwent fluorescent lymphography using NIR imaging, with historical controls. An endoscopic injection of a 1.25mg/mL ICG solution in sterile water was injected into the submucosal layer the day before surgery. In the NIR-ICG group, a larger number of lymph nodes was retrieved: more than 30 lymph nodes were retrieved in 92.5% of patients. The number of lymph nodes dissected from the fluorescent stations was significantly higher, especially at stations 2, 6, 7, 8, and 9, and all metastatic lymph nodes were fluorescent. In this study, non-compliance per patient was defined as the absence of lymph nodes from two or more lymph node stations that were supposed to be harvested, and non-compliance per lymph node was defined as the absence of lymph nodes from the dissected station. The proportion of non-compliance per station was 5.2% for fluorescent stations compared to 27.3% for non-fluorescent stations and 18.5% for historical controls. The proportion of non-compliance per patient was 35% in the NIR group compared to 57.5% in the historical group (*p* = 0.04). The authors supported the hypothesis of a reduction in the non-compliance rate at each draining station. The contamination rate (more than two stations collected that should not have been removed) per patient was 7.5% in the NIR group compared to 2.5% in the sample (*p* = 0.62). Therefore, the authors hypothesized that fluorescence would facilitate node retrieval during pathological examination. This method guarantees an intraoperative assessment of the completeness of the dissection and allows for more accurate diagnosis and pathological staging, corroborating the hypothesis that NIR fluorescent lymphography plays a decisive role in diagnosis and staging. This could possibly lead to the detection of groups of patients in which stage migration (i.e., a change in the prognostic group after reclassification of the extent of the disease) has occurred, which could benefit from adjuvant treatment. With higher sensitivity and specificity for metastatic nodes, the authors suggested that it could be possible in the near future to perform personalized dissection according to individual drainage patterns. In addition, no differences in postoperative complications were observed (24).

A retrospective study was published by the same study group as above, which aimed to evaluate the diagnostic accuracy in lymph node metastasis detection during fluorescent guided lymphadenectomy for T1–4a N0–3 M0 tumors. This was done by assessing the congruency between NIR imaging and histopathological examination of “fluorescent” and “non-fluorescent” lymph nodes. In this study, robotic D1+ gastrectomy was performed for early gastric cancer, and robotic D2 gastrectomy was performed for advanced gastric cancer. Fluorescent nodes beyond the D2 stations were not resected, except at station 14v. The technique showed high accuracy and low false negative rates, with NPVs of 99.3% in non-fluorescent stations, suggesting that changes in lymphadenectomy strategies are possible in order to perform personalized treatments. Therefore, this study suggests that this technique is valid for use in advanced gastric cancer, with the option to minimize the extent of lymphadenectomy, especially in high-risk patients. Notably, the authors expressed concerns regarding false negatives that resulted in acceptable but not satisfactory rates (4.7%) (57).

A Western prospective study analyzed a matched population of patients undergoing robotic gastrectomy with D2 lymphadenectomy (37:37), with and without NIR ICG administration. The number of examined lymph nodes in the control group was 40.1. However, ICG fluorescence failed to demonstrate diagnostic value for metastatic nodes, with sensitivity and specificity values far lower than 90%; in addition, the number of metastatic nodes retrieved was similar between the two groups (58). To obtain high-level evidence, a randomized clinical trial (FUGES-012) was recently conducted in 266 patients with resectable gastric cancer (cT1–4, N0/+, M0). A significantly greater mean number of dissected lymph nodes was registered in the ICG group based on the D2 criteria. A 1.25mg/mL ICG solution in sterile water was injected endoscopically the day before surgery into the submucosa. The authors concluded that ICG-guided equipment may be of great value to newly trained gastric surgeons and can be useful in the dissection of station 14v, considering that metastasis was observed in 33.3% of patients with fluorescent 14v and that 11.6% of the retrieved nodes were metastatic (higher data compared to previous studies). The authors suggested that ICG imaging could significantly reduce the lymph node non-compliance rate for distal and total gastrectomy by performing complete dissection of the stations. Finally, the authors emphasized that only 56.3% of metastatic lymph nodes displayed fluorescence in the study, suggesting that ICG fluorescence could not accurately indicate metastatic lymph nodes and that, from a future perspective of technique diffusion, technological advancements are needed (23).

The FUGES-019 trial, with a design similar to that of FUGES-012, compared subserosal and submucosal injections of ICG. Submucosal injection was performed endoscopically the day before the planned surgery in 133 patients, while the subserosal administration was initiated 20min prior to the beginning of the laparoscopic lymphadenectomy in the other 133 patients. ICG contamination due to mistakes in the administration of ICG during endoscopy or due to intraoperative leakage was very low in both the groups. Twenty minutes after the intraoperative subserosal injection, the luminescence of the D2 stations was comparable to that of the submucosal group. There was no difference in the number of nodes retrieved (*p* = 0.713), and no differences were observed in terms of the nodes collected station by station between the groups. In addition, the non-compliance rates were comparable: 32.3% for the submucosal injection group vs. 33.3% for the subserosal injection group (*p* = 0.860), regardless of the planned surgery (total or distal gastrectomy). The authors concluded that both methods allow for precise staging. Moreover, they analyzed the pattern of diffusion of ICG and found that the dye flowed from the submucosal layer to the serosa through the intermuscular lymphatic network, resulting in a lack of difference in the lymphatic mapping in relation to the site of injection. The authors suggest a specific method for subserosal injection denoted the Huang's subserosal hexa-point maneuver, which consists of the administration of ICG at six specific points along the lesser and greater curvature of the stomach. Ultimately, the cost-effectiveness analysis performed in this study demonstrated how a subserosal injection can be a valid, cheaper solution, particularly considering the workload reduction experienced by endoscopists for tumors other than cT1. In conclusion, the authors did not find any difference in the lymph node-related outcomes between the two methods, with better patient satisfaction and cost effectiveness in favor of subserosal injection; however, they underlined how similar studies should be conducted by analyzing data from neoadjuvated patients (59).

In a cohort study, Zhong et al. presented pooled data from the previously cited FUGES-012 trial (23) and FUGES-019 trial (59). The authors confirmed a higher number of retrieved lymph nodes and a reduction in the non-compliance rate of D2 lymphadenectomies performed using ICG. Moreover, for cT1 and cT2 tumors, the sensitivity for detecting metastatic nodes and the NPV of the non-fluorescent stations were both 100%; lower percentages were reported for advanced T stages, but with documented benefits in terms of the number of nodes retrieved and non-compliance rates. The authors concluded that D1 plus selective imaging-guided lymphadenectomy for cT1-cT2 and D2+ selective imaging-guided lymphadenectomy for cT3-cT4 tumors could be hypothetically included in the current clinical practice guidelines. Interestingly, the reported metastasis rate for stations 10 and 14v was higher than that previously reported, reinforcing the role of image-guided additional lymphadenectomy. However, the authors performed a systematic lymphadenectomy in all patients included in the study, and further studies are needed to assess the feasibility of non-fluorescent lymph node station omission. Furthermore, both the FUGES-012 and FUGES-019 trials collected data from patients who did not undergo neoadjuvant treatment, and different outcomes could be expected from procedures after neoadjuvant treatment due to different node drainage patterns. Therefore, long-term oncological outcomes are still anticipated (60).

To evaluate the safety, effectiveness, and feasibility of ICG in patients with advanced gastric cancer after neoadjuvant chemotherapy (NAC), Huang et al. retrospectively compared 313 propensity score-matched patients in ICG and non-ICG groups. The neoadjuvant treatment consisted of intravenous oxaliplatin and oral fluoropyrimidine S-1. The data showed that ICG was helpful for dissecting a higher number of lymph nodes (40.8* ± *13.7 vs. 31.8* ± *13.5 *p < *0.001), reducing lymph node dissection non-compliance rates (35.1% vs. 51.1%, *p* = 0.027) even in non-responder patients (22.4% vs. 56.2%, *p < *0.001), and reducing blood loss (45.6* ± *19.1 vs. 89.6* ± *89.3mL, *p < *0.001). ICG was particularly useful for dissecting stations 5 and 12a. Moreover, its advantage in terms of node collection was attested in patients with progressive or stable disease after neoadjuvant treatment; non-significant benefits were registered in good responders, even if ICG was merely useful for determining the burden of the nodes and vessels. This is particularly relevant for the accurate staging and determination of the appropriate subsequent treatment in NAC patients (61). Recently, a retrospective study compared the perioperative and long-term outcomes of patients treated with minimally invasive D2+ station 10 total gastrectomy with and without ICG administration. The authors concluded that, with a 54% prevalence of fluorescence at station 10, independent of the tumor epicenters, the lymph node drainage patterns were determined by the areas of tumor involvement, which are unique in every case. The NPV of the method in regard to station 10 dissection was 97%, and the non-compliance rate registered was lower when there was fluorescence at station 10. Interestingly, there was no difference in overall survival, and there was a positive trend in the relapse-free survival between the groups. For patients with negative lymph node metastasis, the relapse-free survival was higher in the ICG group, with marginal significance (*p* = 0.054) (62).

## Evaluation of Parenchymal Perfusion to Avoid Anastomotic Leakage

ICG has also been tested in the assessment of anastomosis quality in both total and subtotal gastrectomy and esophagectomy. Despite recent advancements, anastomotic leakage is a major concern in gastric surgery. The incidence ranges between 2.1% and 14.6% in Eastern countries and up to 26% in Western countries. It is the leading cause of mortality related to the procedure (in a 0–50% range) and leads to poor long-term outcomes (63). To date, subjective evaluations have lacked predictive accuracy for anastomotic leakage. These evaluations include tension-free state, proper tissue apposition, minimal spillage of bowel contents, and adequate blood perfusion (64). Several studies have been conducted to evaluate esophagogastric anastomosis by attempting to quantify the blood supply of the gastric conduit and anastomotic region using ICG, suggesting that fluorescence angiography is useful for reducing anastomotic leakage after esophagectomy (65–68). However, few studies have evaluated esophagojejunal anastomosis using ICG.

Recently, Huh et al. conducted a prospective exploratory study on laparoscopic gastrectomy. Thirty ICG-guided procedures (distal gastrectomy with BI or BII reconstructions, total gastrectomy with Roux-en-Y, and pylorus-preserving gastrectomy) were evaluated with an NIR camera by injecting 2.5–5.0mg/mL of ICG solution immediately after the anastomosis was performed. Clinical and fluorescence assessments were performed with separate perfusion scores. The clinical score based on visual observations (dusky, patchy, pink appearance, pulsatility of the mesenteric vessels, and bleeding cut edges) was high for all procedures, and fluorescence was obtained in 100% of patients, with a gap of visualization of 4.1 *± *3.2 min (range, 2–15) after ICG injection. Although ICG visualization was considered unsuccessful in seven patients (23.3%), no changes in the surgical plan were advised; anastomotic leakage occurred in one patient with high clinical and fluorescence scores. The authors confirmed the feasibility of the technique but advocated further studies to prove its effectiveness and determine the appropriate dosage of injected ICG (69).

Another retrospective study analyzed 100 gastric cancer procedures performed by the same senior surgeon. A subjective evaluation was conducted, followed by NIR ICG fluorescence evaluation. All the patients received a solution dose of 0.5mg/kg. The videos of the procedures were revised, and the evaluation was performed 60 s after the ICG injection. This study demonstrated that the gap in visualization between the two time points of the anastomosis was a useful predictor of anastomotic leaks. Overall, 100% of patients with a defined faint pattern of perfusion developed anastomotic leaks, and one of the four anastomotic leaks had a homogeneous pattern. The authors speculated that the patients’ nutritional status, blood pressure, arteriosclerosis, evaluation of ICG flow speed, or technical issues could explain the leaks, apart from reasons related to intraoperative perfusion. Moreover, they underlined that quantitative measurement of fluorescence is still difficult in clinical practice because of the need for designated software (70). Recently, it has been created the European registry on Fluorescence Image-Guided Surgery aiming to collect high-volume data on the use of NIR fluorescence imaging. To date, most cases in the database involved colorectal procedures. Results from the registry have been presented by Spota et al. (71). Procedures for esophageal and gastric cancer were 21/1240 (1.7%) and 45/1240 (3.6%) respectively. As regard gastric cancer, due to the small size of the sample (27 subtotal gastrectomies and 16 total gastrectomies) subgroup analysis has not been conducted yet. Therefore, the authors advocate for future inclusions among European centers, standardization in terms of equipment or procedural techniques and quantitative analysis to better understand the impact of fluorescence image-guided surgery. In conclusion, more studies are needed to confirm that NIR ICG fluorescence evaluation of anastomosis for gastric cancer surgery can reduce anastomotic leakage and to compare the relevant intraoperative and postoperative parameters (such as variations in blood pressure).

## Perigastric Vessel Navigation

ICG can be useful in vessel navigation as it can identify the shape and origin of small vessels that may not be detected by preoperative imaging studies. Tissue thickness is not considered an issue for near-infrared light when the tissues are 2–4 cm-thick (72, 73). In a recent study, Kim et al. injected a 2.5mg/mL solution of ICG immediately after ligation of the right gastroepiploic vein during 20 consecutives, prospectively enrolled robotic and laparoscopic gastrectomies. The purpose of this study was to assess the presence of an infrapyloric artery, which is to be spared during pylorus-preserving gastrectomy, or an accessory splenic artery emerging from the left gastroepiploic artery in order to prevent inferior polar infarction of the spleen. An infra pyloric artery was identified in 80% of cases, with a procedural time of less than one minute, and the accessory splenic artery, when present, was always easily identified. The authors suggested that ICG could be useful for inexperienced surgeons during infrapyloric dissection and for reducing operative times, blood loss, and the number of unintended injuries (72).

In a retrospective study of 31 patients, Lee et al. described the advantages of ICG technology for detecting accessory left hepatic artery during surgery. The authors evaluated the grade of liver surface fluorescence after an endo-clamp was placed on the artery near the left lobe, assuming that fluorescence mainly depends on the arterial irroration. They then intravenously injected 5mg of ICG diluted in 2 mL of sterile water, and the entire fluorescence of the liver was evaluated. In case of a reduction in the fluorescence of the left lobe, the clamp was removed, and vascularization was reassessed with a new intravenous injection of ICG. According to the accessory artery territories highlighted by NIR ICG, the artery was ligated in 20 patients and preserved in 10 patients, and no differences were observed in terms of intraoperative or postoperative outcomes. The authors concluded that accessory left hepatic arteries could be safely ligated after NIR fluorescence evaluation, avoiding potentially difficult and longer dissections during gastrectomies (73).

## Ongoing Trials

Ongoing trials investigating the role for ICG in lymph node navigation surgery and lymph node mapping include the “multicenter non-randomized phase III study of sentinel node navigation surgery for early gastric cancer” study, which is being conducted by Japanese institutions (UMIN000014401) and aims to evaluate the long-term outcomes of sentinel node navigation surgery for early gastric cancer compared to conventional distal or total gastrectomy. Another is the prospective “Fluorescence Image-Guided Lymphadenectomy in Robotic Gastrectomy (IG-MIG) (NCT03931044)” trial, which compares ICG guided robotic gastrectomy vs. standard robotic gastrectomy, aiming to measure the mean difference in the total number of LNs retrieved during surgery and the percentage of fluorescent nodes detected. The phase II “Indocyanine Green Tracer Using in Laparoscopic Gastrectomy with Lymph Node Dissection (ICGTinLG) (NCT03050879)” RCT aims to evaluate the difference in the number of nodes retrieved between ICG laparoscopic gastrectomy and standard gastrectomy. The phase two “Prospective Clinical Trials on Clinical Outcomes of Indocyanine Green Tracer Using in Laparoscopic Distal Gastrectomy with Lymph Node Dissection for Early Gastric Cancer (NCT04973475)” study has the primary outcome of evaluating the false negative rates of ICG. The “SENORITA 2 Feasibility Study of Sentinel Navigation Surgery in Early Gastric Cancer Patients after Non-curative Endoscopic Resection (NCT03123042)” aims to evaluate the detection rate and false negative rate of sentinel node navigation surgery. The “Indocyanine Green Lymphangiography as a Tool for Improving Lymphadenectomy in Gastric Cancer (NCT04591028)” trial has the primary outcomes of establishing the total number of lymph nodes removed after surgery that are positive for ICG fluorescence, how many are positive for metastasis, and the number of nodes collected per station. Finally, the “iGreenGO Study (NCT04943484)” intends to investigate the “changes in surgical conduct” (CSC) at the moment of intraoperative NIR/ICG technology activation after a D2 lymphadenectomy performed “with the naked eye” (Table 2).

**Table 2:** Ongoing randomized clinical trials.

Ongoing RCTs	Brief description	Primary outcomes
Multicenter non-randomized phase III study of sentinel node navigation surgery for early gastric cancer (UMIN000014401)	Evaluate the long-term outcomes of sentinel node navigation surgery for early gastric cancer compared with conventional distal or total gastrectomy	Postoperative 5-year recurrence free survival (RFS) ratio
Fluorescence Image-Guided Lymphadenectomy Using Indocyanine Green and Near Infrared Technology in Robotic Gastrectomy (NCT03931044)	Evaluate the role of fluorescence imaging during robotic lymphadenectomy for gastric cancer	Mean difference of total number of LNs retrieved during surgery (mean* ± *DS) Fluorescent lymph nodes identification rate (No. %) Accuracy (%; 95% CI)
Prospective Randomized Controlled Trials on Clinical Outcomes of Indocyanine Green Tracer Using in Laparoscopic Gastrectomy With Lymph Node Dissection for Gastric Cancer (NCT03050879)	Predict the positive lymph nodes in gastric adenocarcinoma; guide the scopes in laparoscopic lymph node dissections for gastric adenocarcinoma	Total number of retrieved lymph nodes
Prospective Clinical Trials on Clinical Outcomes of Indocyanine Green Tracer Using in Laparoscopic Distal Gastrectomy With Lymph Node Dissection for Early Gastric Cancer (NCT04973475)	Explore the value of indocyanine green (ICG) in laparoscopic distal gastrectomy with lymph node dissection for early gastric cancer	False negative rate
Feasibility Study of Sentinel Navigation Surgery in Early Gastric Cancer Patients After Non-curative Endoscopic Resection (NCT03123042)	To prove the feasibility of sentinel node navigation surgery (SNNS) in early gastric cancer patients with the risk of lymph node metastasis after endoscopic resection; preparation of the phase 3, multicenter stomach-preserving surgery trial in these patients	Detection rate (%)
Indocyanine Green Lymphangiography as a Tool for Improving Lymphadenectomy in Gastric Cancer (NCT04591028)	Evaluate the safety and added benefit of using the indocyanine green dye (ICG) during surgery	Positive ICG fluorescence lymph nodes Disease positive lymph nodes Number of lymph nodes removed at each lymphatic station
[The iGreenGO Study]. Investigation About the Clinical Value of Indocyanine Green Imaging Fluorescence (NIR/ICG) Technology as a Modifier of Surgeon’s Conduct During Curative Treatment of Advanced Gastric Cancer. Study Protocol for a Western, Observational, Prospective, Multicentric Study	Investigate whether the intraoperative application of NIR/ICG technology is associated with a change in the surgical conduct (CSC) during curative-intent gastrectomy with D2 lymphadenectomy in a cohort of Western patients affected by advanced gastric cancer	Incidence of “changes in surgical conduct” (CSC) at the moment of intraoperative NIR/ICG technology activation after a D2 lymphadenectomy performed “with the naked eye”

## Future Perspectives

The future is bright, and the technological implementation of surgical devices will be inevitable. Almost every producer has developed an integrated NIR ICG system for new optical tools. The use of these systems could prevent the need for upper abdominal incisions, achieve better oncological outcomes, shorten the learning curve, and pursue function-preserving curative gastrectomy. Moreover, integrated technologies capable of quantifying the fluorescence produced by ICG in real time are currently being investigated; presently, only case reports on real-time fluorescence quantification have been reported (74). Studies are in progress regarding the role of ICG in detecting peritoneal carcinomatosis (75). There is also great potential for the implementation of the dye in combination with other molecules, tracers, or monoclonal antibodies that are capable of detecting metastasis and that can be detected by multiple diagnostic tools (MRI, NIR, and multi-modality imaging (FMI) using multiple novel fluorophores) (76, 77); some of these methods have already been tested and are awaiting approval for clinical use. An increasing number of devices are in development with the aim of making the fluorescence quantifiable, overcoming the dual tracer method (78), or in order to assess the quality of perfusion (79). A fascinating angle on new technologies that could well integrate with ICG fluorescence imaging is offered by studies on hyperspectral imaging (HSI) and confocal laser endomicroscopy (CLE). HSI is a method able, through real time analysis of tissues’ chemical properties, to potentially detect early mucosal lesions, to measure anastomotic perfusion, to assess resection margins and extent of node dissection in combination with deep learning models (80). CLE is, on the other hand, an optical imaging modality that provides an *in vivo* histopathological assessment of the mucosa, for example in terms of quality of perfusion. (81)

## Conclusions

With relatively no collateral effects and without significantly compromising operative times, ICG fluorescence imaging applications are carving out a role in future studies on gastric resections. The most effective application of this tool has been debated. With regard to sentinel node mapping, ICG guidance is still in the preliminary phase. Steps are being taken to invest in the concept of basin dissection instead of node picking, collecting more nodes to reduce the risk of false negatives, and developing more precise techniques for intraoperative pathological examinations. Nevertheless, high-quality evidence with which to provide a strong statement on the topic is still lacking. As previously mentioned, especially for the Western population, due to the low incidence of metastasis for early gastric cancer, thousands of cases may be required to obtain high-quality data. Indocyanine green fluorescence appears to be helpful for the dissection of more lymph nodes, even during planned lymphadenectomies. Preliminary evidence suggests that NIR with ICG can help surgeons assess the completeness of D1, D1+, or D2 lymphadenectomy and define the boundaries of the dissection, all while highlighting the anatomical landmarks and fluorescent stations. This could be particularly useful for training young surgeons to perform minimally invasive procedures to retrieve enough nodes and perform adequate lymphadenectomies.

The impact of ICG on non-compliance and contamination rates must be investigated. Therefore, by systematically exploring beyond the secondary level stations under the guidance of ICG fluorescence, we could better define the real locations of stations such as station 14v or even 13, and consider, in particular contexts (such as in large tumors of the antrus or in the lower part of the great curvature, with serous invasion, or with station 6 confirmed nodal involvement), the search and removal of such stations. The use of ICG could also lead to less extended, tissue-sparing surgeries and perhaps more precise staging, especially in T1 or T2 tumors, by assessing their nodal state and detecting micrometastasis, thus allowing the evaluation of any adjuvant treatment. From this standpoint, apart from identifying the first draining node, which is the protocol in the sentinel node scenario, identifying the last draining node may have added value in achieving lymph nodal R0 resections.

Based on the suggestions offered by the authors of the papers presented in this review, it can be speculated that D1+ or D2 lymphadenectomy is not always perfect or even necessary. Moreover, according to the findings of this review, patients with advanced gastric cancer who underwent surgery after neoadjuvant chemotherapy could benefit from the use of NIR with ICG. In fact, it seems capable of defining lymphatic drainage in progressive or stable disease after neoadjuvant treatment. Standardization and objective measures are required for the development of real-time software. Once the technique is standardized, surgeons will speculate on the usefulness of the tool and analyze the direct and indirect costs of its use. Increasing evidence is still needed to support this trend due to the multiple benefits offered.
